# Dragon exploration system on marine sponge compounds interactions

**DOI:** 10.1186/1758-2946-5-11

**Published:** 2013-02-16

**Authors:** Sunil Sagar, Mandeep Kaur, Aleksandar Radovanovic, Vladimir B Bajic

**Affiliations:** 1 King Abdullah University of Science and Technology (KAUST), Computational Bioscience Research center, Thuwal, 23955-6900, Saudi Arabia

**Keywords:** Sponge compounds interactions, Natural products, Text-mining, Information integration, Knowledge base

## Abstract

**Background:**

Natural products are considered a rich source of new chemical structures that may lead to the therapeutic agents in all major disease areas. About 50% of the drugs introduced in the market in the last 20 years were natural products/derivatives or natural products mimics, which clearly shows the influence of natural products in drug discovery.

**Results:**

In an effort to further support the research in this field, we have developed an integrative knowledge base on Marine Sponge Compounds Interactions (Dragon Exploration System on Marine Sponge Compounds Interactions - DESMSCI) as a web resource. This knowledge base provides information about the associations of the sponge compounds with different biological concepts such as human genes or proteins, diseases, as well as pathways, based on the literature information available in PubMed and information deposited in several other databases. As such, DESMSCI is aimed as a research support resource for problems on the utilization of marine sponge compounds. DESMSCI allows visualization of relationships between different chemical compounds and biological concepts through textual and tabular views, graphs and relational networks. In addition, DESMSCI has built in hypotheses discovery module that generates potentially new/interesting associations among different biomedical concepts. We also present a case study derived from the hypotheses generated by DESMSCI which provides a possible novel mode of action for variolins in Alzheimer’s disease.

**Conclusion:**

DESMSCI is the first publicly available (http://www.cbrc.kaust.edu.sa/desmsci) comprehensive resource where users can explore information, compiled by text- and data-mining approaches, on biological and chemical data related to sponge compounds.

## Background

Natural products are chemical compounds that originate from living organisms and play a major role in the drug discovery and development process. The importance of natural products in drug discovery has been discussed in several reviews and reports [1-5]. About 200,000 natural compounds are currently known [6]. The chemical diversity of natural compounds, especially the diversity of scaffolds and the large number of chiral centers represent a basis for their use in drug therapy. About 50% of the drugs introduced in the market during the last 20 years are derived directly or indirectly from natural products [7]. A total of 1,184 new approved drugs have been identified covering all diseases/countries/sources in the years 01/1981-06/2006. Out of these, only 30% were synthetic in origin, which demonstrates the influence of natural products/derivatives/natural product mimics on drug discovery process [8].

The marine organisms adapted to unusual conditions of higher salt content, low or zero light, unusually low or high temperature and pressure, have offered a number of lead bioactive molecules with unique novel structures and distinct biological activities. Marine sponges have been considered a valuable source of bioactive molecules with different pharmacological activities. Sponges produce a wide array of secondary metabolites ranging from derivatives of amino acids and nucleosides to porphyrins, terpenoids macrolids, sterols, and others. Reports of isolation and identification of natural products from marine sponges are being published since early 1950’s. The isolation and identification of spongothymidine and spongouridine from the Caribbean sponge *Tethya crypta*[9-11], led to the discovery of close analogues, cytosine arabinoside or Ara-C, as a potent antileukemic agent and adenine arabinoside or Ara-A, an antiviral compound, as commercial drugs. The sponge-derived apoptosis-inducing lead compounds that have potential use in cancer treatment have been described in a recent review [12].

The investigation of several sponge compounds in clinical trials (Table 1) for various diseases proved the significance of sponges as an important source organism in the drug discovery [13]. Due to the importance of the sponges and their bioactive compounds, the voluminous research work in this area has generated a plethora of published scientific reports. A query in PubMed database (http://www.ncbi.nlm.nih.gov) using keywords “porifera OR sponge OR sponges” retrieved 16,023 abstracts (31 December, 2012).

**Table 1:** Sponge derived bioactive molecules in clinical and preclinical trials

**Name**	**Sponge**	**Disease**	**Status**
Discodermolide	*Discodermia dissoluta*	Cancer	Phase I
E7389	*Lissodendoryx sp*	Cancer	Phase III [14]
HTI-285 (hemiasterlin derivative)	*Cymbastella sp*	Cancer	Phase II
KRN-7000	*Agelas mauritianus*	Cancer	Phase I
Peloruside A	*Mycale hentscheli*	Cancer	Preclinical
Salicylihalimides A	*Haliclona sp*	Cancer	Preclinical
Laulimalide	*Cacospongia mycofijiensis*	Cancer	Preclinical
Variolins	*Kirkpatrickia variolosa*	Cancer	Preclinical
Dictyodendrins	*Dictyodendrilla verongiformis*	Cancer	Preclinical
Manoalide	*Luffariaella variabilis*	Antipsoriatic	Phase II/discontinued
Bengamide derivative	*Jaspis sp*	Cancer	Phase I/discontinued
Girolline	*Pseudaxinyssa cantharella*	Cancer	Phase I/discontinued

The significance of sponge bioactive compounds in the drug discovery process and the lack of the public resource with the relevant information, motivated us to develop Dragon Exploration System on Marine Sponge Compounds Interactions (DESMSCI) as a public web-based knowledge base that integrates and allows exploration of information about sponge natural products and their potential biological and chemical associations. It is compiled from the published literature available in PubMed and complemented by the information from 25 other resources (Table 2). DESMSCI is the first publicly available, fully searchable, web-enabled knowledge base, where the information related to the sponge natural products can be explored at molecular levels, providing insights into related or affected human genes and proteins, diseases, and associated biological pathways, as well as potential mutual links among these entities. The information is generated by Dragon Exploration System (DES), a biomedical text mining and data mining system. DES has been previously used as the engine in creation of a number of topic-specific knowledge bases [15-18].

**Table 2:** External databases used for data integration

**Database**	**URL**	**Number of records**
Chemical Entities of Biological Interest (ChEBI)	http://www.ebi.ac.uk/chebi/init.do	38,580
ChEBI Ontology	http://www.ebi.ac.uk/chebi/	29,974
Enzyme	http://ca.expasy.org/enzyme/	5,418
Gene	http://www.ncbi.nlm.nih.gov/gene	892,7911
Functional association data/networks (GeneMania)	http://genemania.org/	21,084
GO	http://www.geneontology.org/	34,940
GOA	http://www.ebi.ac.uk/GOA/	11,300,749
HUGO Gene Nomenclature	http://www.genenames.org/	35,795
Human Major Histocompatibility Complex	http://www.ebi.ac.uk/imgt/hla/	6,939
Immunoglobulins and T cell receptors nucleotide sequences	http://www.imgt.org/IMGTlect/	156,529
Interpro	http://www.ebi.ac.uk/interpro/	21,749
Oxford Human Mouse grid	http://www.informatics.jax.org/	17,834
Pfam-A	http://pfam.sanger.ac.uk/	12,273
Pfam-B	http://pfam.sanger.ac.uk/	233,174
Pfam-Seed	http://pfam.sanger.ac.uk/	12,273
PRINTS	http://bioinf.man.ac.uk/dbbrowser/PRINTS/	2,050
Prosite	http://www.expasy.org/prosite/	2,247
Prosite Documentation	http://www.expasy.org/prosite/	1,621
REBASE	http://rebase.neb.com/rebase/rebase.html	5,020
RefSeq	http://www.ncbi.nlm.nih.gov/RefSeq/	18,236,994
UniProtKB/Swiss-Prot	http://www.uniprot.org/	53,1473
Taxonomy	http://www.ncbi.nlm.nih.gov/entrez/query.fcgi?db=Taxonomy	817,120
UniProtKB/TrEMBL	http://www.uniprot.org/	16,504,022
Unigene	http://www.ncbi.nlm.nih.gov/entrez/query.fcgi?db=unigene	2,652,777
Uniprot: UniProtKB/Swiss-Prot, UniProtKB/TrEMBL	http://www.uniprot.org/	17,035,495
	Total Records	76,644,041

### DESMSCI Resource

DESMSCI knowledge base is a resource that is compiled using as its primary engine, the KAUST-customized version of Dragon Exploration System (DES). Original DES is a proprietary text-mining and data-mining tool from OrionCell (http://www.orioncell.org). The titles and abstract of PubMed records are downloaded and indexed using several dictionaries. Each of the dictionaries consists of curated names and symbols and their variants customary for the specific types of entities (Table 3). For the data integration purposes the MRS [19] was deployed. Data was downloaded from 25 sources and indexed, producing more than 76,000,000 records (Table 2). The data are linked to annotated terms on-demand basis.

**Table 3:** Dictionaries used for text mining during creation of MSCI

**Dictionary**	**Number of terms**	**Number of terms found**
Disease concepts	84,282	3,946
Human genes and proteins	269,908	2,449
Mode of action	427	284
Pathways	3,717	58
Sponge Compounds	3,050	517
Total: 5 dictionaries	Total: 361,384	Total: 7,254

DESMSCI database was built on 31 December, 2012, with a document collection consisting of 16,023 abstracts downloaded from PubMed using “porifera OR sponge OR sponges” as a query. Annotation terms we used (Table 3) were from the following dictionaries: “Sponge compounds”, “Human genes and proteins”, “Mode of action”, “Pathways”, and “Disease concepts”. The dictionary of “Sponge compounds” contains manually curated 3,050 sponge compounds (including synonyms), compiled from the published literature. The dictionary of genes and proteins contains 269,908 variants of entities covering the names, symbols, aliases, previous names and previously used symbols of human genes and proteins. The DES engine performed annotation and created indexes of terms, terms pairs and clustering of PubMed articles. Finally, DESMSCI web interface was built by using DES customizable modules. Data integration to local MRS installation was implemented by using SOAP based MRS client [http://search.cpan.org/dist/MRS-Client/].

The details about the methods applied by DES, how to use the knowledge base and other relevant details are provided in the documentation (http://www.cbrc.kaust.edu.sa/desmsci/desmsci.pdf). The accuracy of the integrated data was evaluated earlier in Sagar et al. [18] in terms of precision (ability to identify the correct entities of a specific type in PubMed abstracts relative to all identified entities of that type) and recall (the ability to identify correct entities of a specific types present in the abstracts relative to all entities of that type present in the abstracts) and were found to be in the range of 81%–100% for different categories, with an average F-measure of 92.9%. In another report from our group, precison and recall were in ranged from 78%–99% and 87%–100% [15]. A brief comparison with PolySearch [20], a web based text-mining system, has also been reported [18]. However, these accuracy assessments can only be used as a guide and not as a claim of absolute accuracy of the system.

### Generation of text-mined and data-mined reports

**Figure 1:**
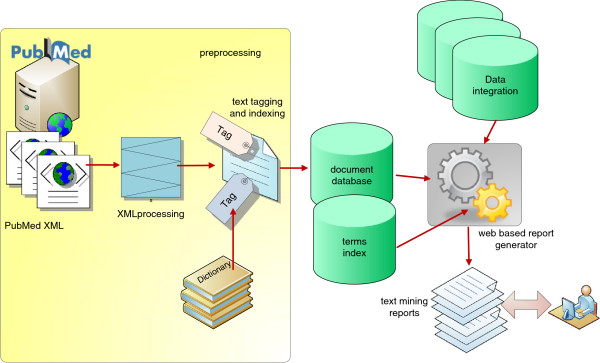
An overview of the text mining methodology.

DESMSCI contains query engine for the processing of user’s queries. The system uses a set of abstracts (obtained as a result of querying PubMed) and mines these abstracts for the presence of terms listed in the curated dictionaries (Table 2). Therefore, the concepts present in the dictionaries are mapped to only that set of abstracts. These mapped concepts are further used to generate results that users see. It is important to understand the work-flow of the process and to know how the system works (Figure 1). The results of queries to DESMSCI are presented in the form of tables or networks which enable users to view the associations of a chemical compound of interest with other biological concepts, such as genes, proteins, diseases, pathways, etc. The links to other external databases are provided that enables users to explore the entity of interest in several external resources (to study structure, function etc.). DESMSCI also provides search options through the use of simple logical operators "AND", "OR" and "NOT" that further allow users an easier and direct access to each of the reports. Simple association networks can be generated for each of the identified concepts. A user-chosen concept represents a center node for such a network. This concept is connected to other concepts based on the co-occurrence metrics. Edge connects two concepts co-occurring in same abstracts and carries a weight representative of the number of those abstracts. Networks can be expanded or shrunk by selecting various weight thresholds and subsets of dictionaries. This is a convenient and efficient method to explore huge amounts of literature data in shorter time and to visualize the important associations among different terms in an easy to follow graphical representations. This functioning of the text-mining modules is based on similar concepts as used in Bajic *et al*. [21].

### Hypothesis generation

Hypothesis generation is one of the most useful features in DESMSCI. It allows users to infer potentially new/interesting relationships among different concepts. The module is based on Swanson’s ABC model [22]. It would be very difficult if not impossible to manually extract the associations between the concepts (which do not appear in the same document), to infer potentially new hypotheses, especially with the large amount of available concepts and literature. DESMSCI allows for the inspection of automatically generated hypotheses and their validity by retrieving the PubMed document(s) related to the concepts linked through the hypothesis. The initial hint that the association between the two concepts may be a candidate for a hypothesis appears in the case when there is no connection (co-occurrence in the same PubMed entry) of the concepts found in the analyzed set of PubMed entries. DESMSCI provides for the further inspection if the same two concepts co-occur in the same PubMed entry by querying the whole PubMed (22,000,000+ entries). If no PubMed document containing both of the terms is found, it suggests a possible new association between such concepts, a hypothesis for further exploration.

## Case studies

### Variolins for preventing neurodegeneration in Alzheimer’s disease

**Figure 2:**
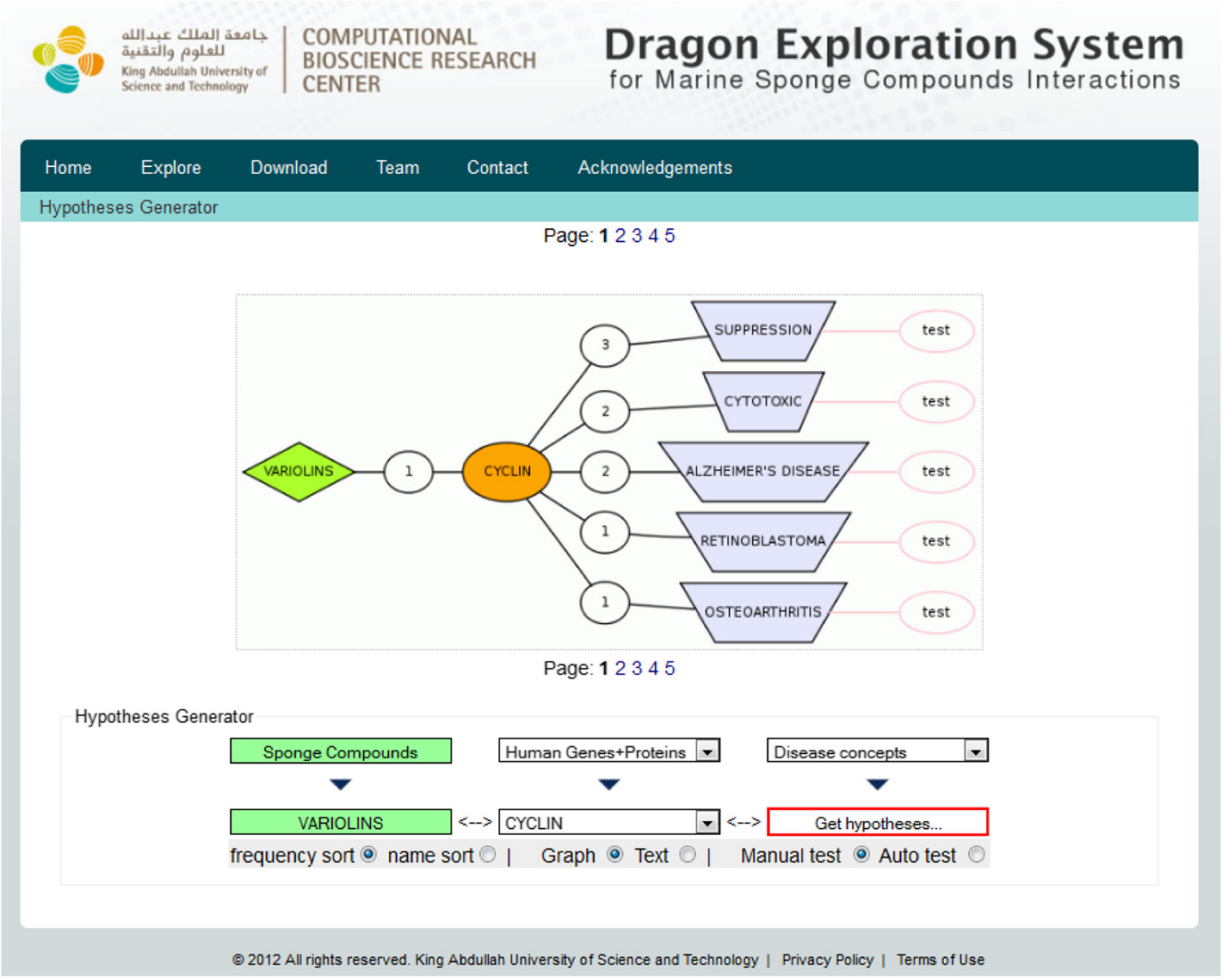
**Hypotheses describing links between ‘Variolins’ and ‘Disease concepts’ through ‘Cyclin’ in human genes and protein dictionary.** The interactions are based on co-occurrence of two entities in relevant PubMed literature.

According to hypothesis generated by DESMSCI (Figure 2), the term ‘variolins’ from “Sponge compounds” dictionary is linked to the term ‘cyclin’ from “Human Genes and Proteins” dictionary through one abstract, while ‘cyclin’ is further linked to ‘Alzheimer’s disease’ (AD) from “Disease concepts” dictionary via two abstracts. On searching the whole PubMed using the “test” button for the co-occurence of terms ‘variolins’ and ‘Alzheimer’s disease’, no PubMed record with such two terms was available. Thus, it was not possible to establish a link between variolins and AD. We further explored the underlying biology to search for any indirect link between these two concepts and to check the validity of the hypothesis generated.

Variolins are anti-tumor marine alkaloids isolated from a rare Antarctic sponge *Kirkpatrickia Varialosa* in 1994 [23]. Variolin-B (VAR-B) was most cytotoxic among the four compounds isolated from sponge and later derivatives of variolins were synthesized to enhance aqueous stability as well as their anti-cancer activities [24]. The studies on a derivative (dVAR-B) of VAR-B showed that variolins are CDK (cyclin-dependent kinases) inhibitors and induce apoptosis via p53 independent mechanism [25]. Cyclins, CDKs and cyclin-dependent kinase inhibitors (CKIs) are cell cycle regulatory proteins that control cell cycle transition from one phase to another (G1, S and G2). Cyclins and CDKs form heterodimers leading to progression or inhibition of cell cycle and these pairs are further inhibited or inactivated by small CKI peptides. The cell cycle deregulation leads to neurogeneration. In neurons, cell cycle normally does not progress beyond G1 phase checkpoint, but in AD, cell cycle progresses to G2 phase that leads the cell to death [26], and also drives the formation of neurofibrillary tangles and amyloid plaques [27-29]. This leads to neurodegeneration which is a characteristic phenotype linked to AD [30]. CDKs (CDK1, CDK2, and CDK5) have been associated with tau hyperphosphorylation, amyloid precursor protein processing, and apoptosis due to the cell cycle deregulation in AD [31]. Therefore, the agents that block cyclins or CDKs may further block neurodegeneration in AD patients [32]. Thus, we can propose a hypothesis that variolins, being inhibitors of CDKs, could block neurodegeneration in AD. Consequently, this potential activity of variolins could be tested for its effects in AD.

### Furospongolide as an angiogenesis blocker

**Figure 3:**
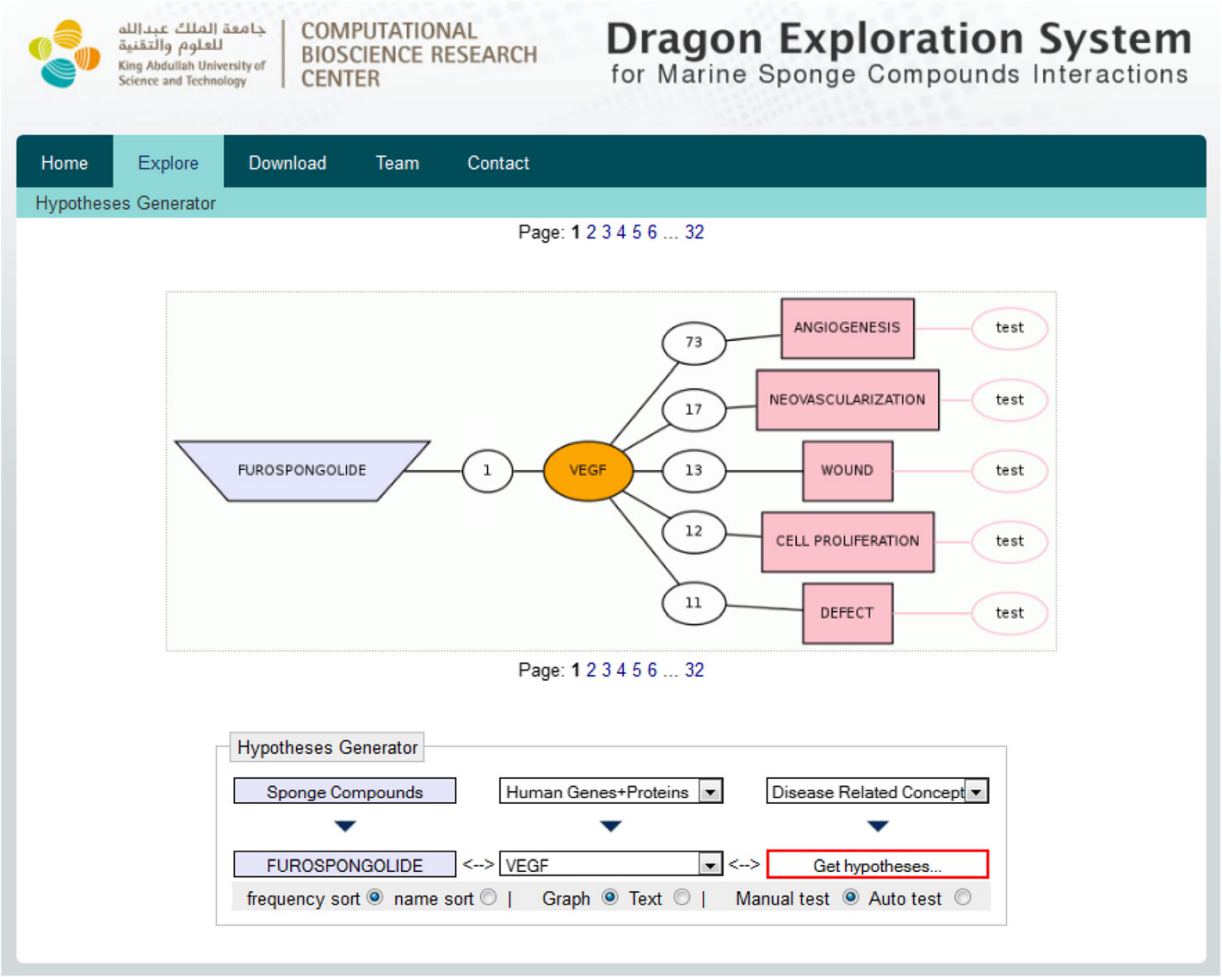
**Potential hypothesis linking ‘Furospongolide’ to ‘Angiogenesis’ via ‘Vascular endothelial growth factor’.** The interactions are based on co-occurrence of two entities in the relevant PubMed records.

**Figure 4:**
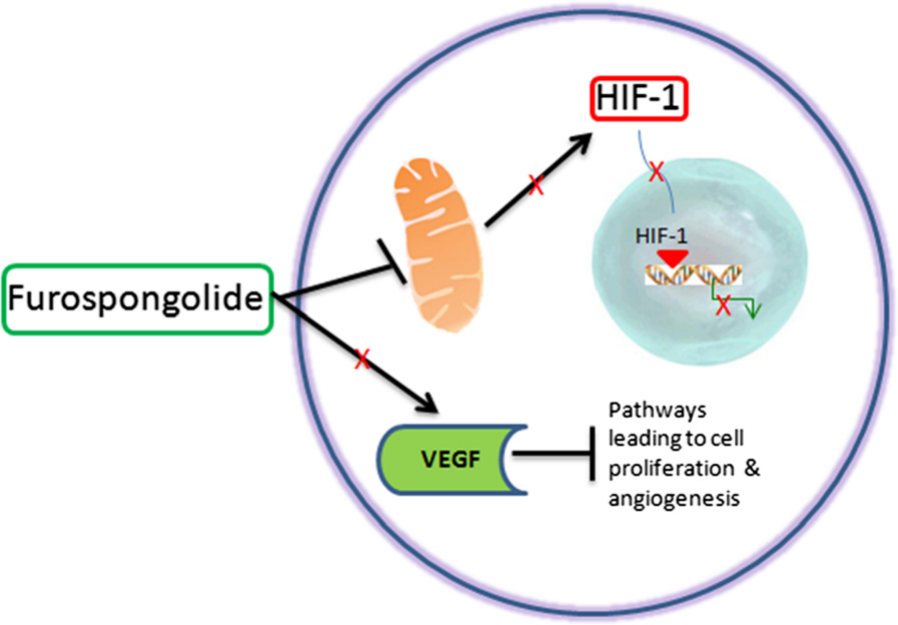
Possible mechanisms of action of Furospongolide in a tumor cell.

The term ‘furospongolide’ from “Sponge compounds” dictionary is linked to vascular endothelial growth factor ‘VEGF’ from “Human genes and proteins” dictionary through one PubMed record, while ‘VEGF’ is further linked to ‘angiogenesis’ from “Disease concepts” dictionary via 73 PubMed records. Thus, we have an indirect link between ‘furospongolide’ and ‘angiogenesis’ (Figure 3). Testing the co-occurrence of these two terms in the whole PubMed retrieved no results. The in-depth analysis of the mechanism of action of furospongolide reveals that this compound is an inhibitor of HIF-1 via a mitochondrial respiratory chain mechanism, where it exerts an inhibitory effect on mitochondrial respiratory chain complex I without any effect on other complexes. Furospongolide inhibits HIF-1 by suppressing tumor cell respiration via NADH-ubiquinone oxidoreductase (complex I)-mediated mitochondrial electron transfer [33]. The inhibition of HIF-1 further leads to suppression of expression of several genes that are activated by binding of HIF-1 on their promoters [34,35]. Furospongolide also inhibits HIF-1 targeted vascular endothelial growth factor (VEGF). VEGF is a key stimulant of tumor angiogenesis (formation of new blood vessels that feeds cancerous cell growth) and suppression of VEGF normally blocks the angiogenesis in tumor cells [36]. Thus, the proposed hypothesis is that furospongolide could be used as a drug to block angiogenesis in tumors (Figure 4).

## Conclusions

DESMSCI is the first publicly available knowledge base where users can explore various types of information about sponge natural products at chemical, biological and molecular levels. Hypothesis generation is an important component of this system and it can help researchers to develop new ideas and test them by using the available literature and other information repositories. We hope that this knowledge base will serve as a useful complement to the existing public resources and for researchers involved in natural products’ research at any level across different disciplines.

### Future directions

DESMSCI will be updated every six months and the information from all new studies published in that period will be incorporated. As the number of concepts grows with new incoming literature, the dictionaries will also be further curated and expanded. The improvement in the quality of dictionaries will certainly enhance the accuracy of the knowledge base. The comments obtained from the users will also help to improve the functionality of DESMSCI.

## Abbreviations

DES: Dragon Exploration System; DESMSCI: Dragon Exploration System on Marine Sponge Compounds Interactions; VAR-B: Variolin-B; CDK: Cyclin-dependent kinases; CKI: Cyclin-dependent kinase inhibitors; AD: Alzheimer’s disease; VEGF: Vascular endothelial growth factor; HIF: Hypoxia inducing factor

## Competing interests

VBB and AR are partners in the OrionCell Company whose product, Dragon Exploration System, has been used in the creation of DESMSCI. Other authors declare no conflict of interest.

## Authors’ contribution

SS and MK conceptualized the study and analyzed the results. SS, MK, AR and VBB wrote the manuscript. AR and VBB developed the DES system. All authors read and approved the final manuscript.
